# Statin-associated weakness in myasthenia gravis: a case report

**DOI:** 10.1186/1752-1947-4-61

**Published:** 2010-02-20

**Authors:** Michael J Keogh, John M Findlay, Simon Leach, John Bowen

**Affiliations:** 1 Department of Stroke Medicine, United Lincolnshire Hospitals Trust, Lincoln County Hospital, Lincoln, LN2 5QY, UK

## Abstract

**Introduction:**

Myasthenia gravis is a commonly undiagnosed condition in the elderly. Statin medications can cause weakness and are linked to the development and deterioration of several autoimmune conditions, including myasthenia gravis.

**Case presentation:**

We report the case of a 60-year-old Caucasian man who presented with acute onset of dysarthria and dysphagia initially attributed to a brain stem stroke. Oculobulbar and limb weakness progressed until myasthenia gravis was diagnosed and treated, and until statin therapy was finally withdrawn.

**Conclusion:**

Myasthenia gravis may be underappreciated as a cause of acute bulbar weakness among the elderly. Statin therapy appeared to have contributed to the weakness in our patient who was diagnosed with myasthenia gravis.

## Introduction

Myasthenia gravis (MG) is characterised by fatigable muscle weakness and has an incidence of only 1 in 5 to 10,000 people [1]. Autoimmune myasthenia gravis, often in association with thymus hyperplasia or thymoma, can affect young adults. However, it is now recognized that myasthenia gravis is actually more prevalent in middle-aged and older groups than younger age groups [2]. In elderly patients, bulbar presentation is common [3] and often mislabeled as a stroke [4] leading to poorer rates of survival [5].

Statins (inhibitors of 3-hydroxy-3-methyl-glutaryl-CoA reductase) lower the incidence of cerebrovascular disease and coronary heart disease. Statin use has increased dramatically over the last decade, with a four-fold increase from 1996 to 1998 [6].

Although generally well-tolerated, statins may have primary care discontinuation rates of up to 30% [7] due to their side effects such as headache, myalgia, paraesthesia, and abdominal discomfort.

Here, we report a case of acute myasthenia gravis presenting in a 60-year-old Caucasian man whose condition deteriorated until immunosuppressive therapy was commenced and statin therapy was withdrawn.

## Case presentation

A 60-year-old Caucasian man of British origin was admitted to our hospital in September 2007 following acute onset of dysarthria and dysphagia. He was diagnosed with diabetes mellitus and hyperlipidaemia three months prior to presentation.

He had no visual disturbance or sensorimotor symptoms in his limbs or torso on presentation. He was commenced on gliclazide, ramipril and aspirin when he was diagnosed with diabetes and hyperlipidemia 3 months earlier. He was also started on simvastatin at that time, but this was stopped following the development of proximal muscle weakness, myalgia, and an elevated creatine kinase (CK) of 2599 (normal: <200), which all resolved upon the termination of this medication. Gliclazide, ramipril and aspirin, however, were continued.

Aside from the finding of mild dysarthria, examination revealed that our patient had no remarkable conditions. Results of routine haematology, biochemistry, thyroid function tests, and creatine kinase were also unremarkable. His serum cholesterol was 6.1 mmol/L and his random blood glucose was 11.2 mmol/L.

An initial diagnosis of a brain stem stroke was considered, so dipyridamole and atorvastatin were added to his medication four days after his admission to our hospital. Meanwhile, a computed tomography (CT) brain scan showed that he had no obvious infarct.

Our patient remained stable over the next few days with a mild dysarthria and dysphagia (tolerating soft food), but no other symptoms or signs were noted.

One week after his admission to our hospital, his dysarthria and dysphagia worsened. Bilateral fatigable ptosis, diplopia, fatigable weakness of his neck flexion, and shoulder abduction were noted for the first time. A previously planned cranial magnetic resonance brain scan was thus cancelled.

Edrophonium testing demonstrated a dramatic transient improvement in his dysarthria, and a diagnosis of myasthenia gravis with high titre anti-acetylcholine receptor antibodies was confirmed. A serum immunoglobulin assay revealed an IgA level of <0.05 g/L. He was noted to have normal IgG and IgM, and no paraprotein band.

Our patient was then commenced on treatment with pyridostigmine. He was also started on incrementally increasing prednisolone every other day. Regular monitoring of his respiratory function was also initiated.

His respiratory function worsened over the next 3 days. His spirometry also deteriorated. He developed a new fatigable diplopia and an inability to stand from a low squat position, together with increasing neck and proximal limb weakness.

In view of his deteriorating state, intravenous immunoglobulin therapy (IVIg) was commenced. Following immunological advice regarding his low IgA titre, it was decided to use Vigam Immunoglobulin (2 g/kg over the next 4 days), which did not result in any adverse effect.

No objective gains were noted over the subsequent week, and a repeat CK yielded a result of 842 mmol/L. His atorvastatin medication was then stopped two weeks after it had been introduced. Following this, our patient showed significant improvement in ptosis, a resolution of diplopia, and improved neck, shoulder, and elbow power. His ability to stand from a low squat position returned, and significant spirometric improvements were also seen.

**Figure 1:**
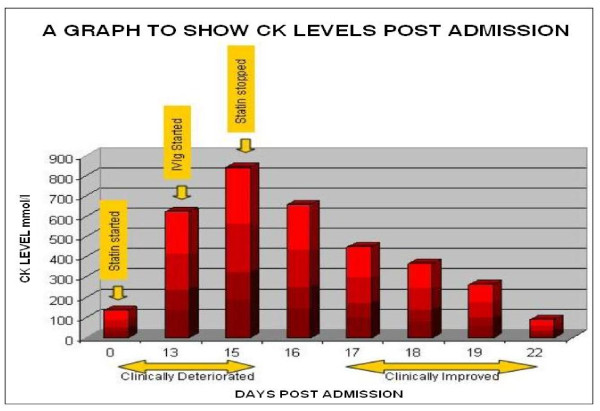
**This image of a graph shows creatinine kinase readings during admission, and a correlation to clinical progress**.

His CK readings fell over this period and returned to normal levels one week after the cessation of his statin medication (Figure 1).

Our patient remained stable until two weeks later when, just prior to a planned discharge, a further deterioration and unresponsiveness to a second course of IVIg necessitated respiratory and nutritional support, intensive care, and plasma exchange.

Following prolonged treatment, his muscle strength improved and he returned to independent living at home four months after his admission to our hospital. His gastrostomy feeding tube and tracheostomy were removed 10 months after he was discharged from our hospital.

## Discussion

Myasthenia gravis has an incidence of only 1 in every 5 to 10,000 people and is potentially fatal. A recent study suggests that 2.2% of patients admitted with myasthenia gravis overall died during admission [8], and that the risk could be reduced by 69% if the patient is under the care of a neurologist. It is thus important not to readily dismiss the condition and that appropriate referrals are made.

The actual incidence of statin-exacerbated myasthenia is unknown, and only a handful of reports of statin-associated myasthenia gravis have ever been described [9-11].

Out of 6 published case reports, only 5 patients were noted to have some degree of recovery and only one patient had a complete recovery upon termination of statin therapy [11].

How statins could appear to exacerbate MG is unclear. It is possible that the mechanism actually reflects a "double hit" phenomenon of defective neuromuscular transmission secondary to antibody-mediated post-synaptic acetylcholine receptor dysfunction in combination with a statin-induced myopathy.

The clear development of a statin myopathy with simvastatin treatment prior to the onset of myasthenia in our patient is consistent with the possibility of a second (atorvastatin- induced) myopathy coalescing with the onset of myasthenia gravis. The symptomatic improvement that followed his withdrawal from atorvastatin treatment resulted from the resolution of this statin myopathy.

We also considered other potential causes of deterioration such as sepsis, steroid-induced worsening of MG, steroid myopathy, and cholinergic crisis, but we considered their development less likely based on clinical grounds.

We cannot rule out completely the possibility that the worsening of our patient's MG simply reflected a progression of his MG. However, the clinical course of his condition, as well as the statin-induced proximal limb pain and weakness (without bulbar features) he experienced prior to his presentation, raises at the very least the possibility that a component of his initial deterioration was statin-related.

Similarly, we note that his improvement could have reflected the immunosuppressive effects of therapy for his MG rather than the withdrawal of his atorvastatin treatment. It seems probable, however, that both factors played a significant role in the improvement of his clinical state.

The development of other autoimmune disorders such as dermatomyositis [12], polymyalgia rheumatica, vasculitis [13], and Lupus-like syndrome [14] upon initiation of statin therapy [13] raises the possibility that in predisposed individuals, statins may precipitate an immunological trigger that is analogous to penicillamine-induced MG [15] although clearly different in temporal respect. However, given the paucity of reports and the widespread use of statins, the possibility of chance association cannot be excluded still.

## Conclusion

Myasthenia gravis is a potentially fatal condition that should be considered in elderly patients with bulbar symptoms. Statin medication should be introduced cautiously and considered as a potential cause or precipitant of worsening muscle strength in patients with myasthenia gravis.

## Abbreviations

CK: creatinine kinase; CT: computed tomography; IVIg: intravenous immunoglobulins; MG: myasthenia gravis.

## Consent

Written informed consent was obtained from the patient for publication of this case report and any accompanying images. A copy of the written consent is available for review by the Editor-in-Chief of this journal.

## Competing interests

The authors declare that they have no competing interests.

## Authors' contributions

MJK reviewed the patient's clinical data, performed the literature search, and wrote the initial draft of the manuscript. JMF, SL and JB reviewed the initial draft and finalized the manuscript. All authors read and approved the final manuscript.
